# Kali Bichromicum*The above is a summary of an article written by Dr. Lippe, and published in *The Hahnemannian Monthly* of August, 1865. We think it will bear republishing.

**Published:** 1887-06

**Authors:** Ad. Lippe

**Affiliations:** Philadelphia


### KALI BICHROMICUM.*

AD. LIPPE, M. D., PHILADELPHIA.

Kali bichromicum is often the only remedy in *morbilli,* especially if the cough, expectoration, and the other catarrhal symptoms correspond with the characteristic symptoms of this medicine. The eruption found under Kali bichromicum is a "solid eruption like measles." The catarrhal symptoms, itching of the canthi: after smarting, itching, and watering frequently during the day and morning, agglutination during the previous

---

*The above is a summary of an article written by Dr. Lippe, and published in *The Hahnemannian Monthly* of August, 1865. We think it will bear republishing.

days. (Pulsatilla has a similar symptom, but the itching of the eyes compels the patient to rub the eyes incessantly; and if this symptom prevails, Puls. will quickly relieve it.) 1. Dr. Drysdale gives as corresponding remedies, Bell., Cop., Guaj., Merc.—Guaj. is new—he has omitted *Aconit.*, *Pulsat.*, Ant. or Bry., Rhus, Sulph., etc.

The catarrhal symptoms of the nose are, considerable flow of water from the nose, subsequently becoming acrid, burning the upper lip and excoriating the nostrils; a small quantity of acrid mucus is discharged from the nose; which causes burning in the septum; nose full of thick mucus, profuse discharge of thick, clear mucus from the nose; when this ceases it is followed by pain from the occiput to the sinciput. These symptoms show the general applicability of Kali bichr. in morbilli. We will now give some of the special characteristic indications for its use in the various forms and individual cases of this disease. We find stiff, green-colored masses of an offensive smell are discharged from the nose. Kali bichr. differs in this respect from Pulsatilla, which has long-continued coryza, with blowing from the nose of yellowish-green mucus, smelling bad. The discharge of Kali bichr. is more stiff, more compact, more green and offensive than that of Pulsatilla. We find that the soreness of the nostrils under Kali bichr. consists in an ulceration, a formation of small burning ulcers, first on the right side, later on the left side, within the nostrils. Pulsatilla has sore, ulcerated nostrils, *i. e.*, the edges of the nostrils are ulcerated. The most important indication for Kali bichr. in measles is the croupy cough accompanying this disease. We find roughness in the larynx, with hoarseness. Suddenly in the evening great hoarseness and roughness of the voice—rough, hoarse voice. He is deprived of his voice; loud, rattling cough for five minutes at a time, with retching and *expectoration of tough mucus, so viscid that it can be drawn to the feet in strings.* During sleep wheezing and rattling in the chest, which can be heard at a distance—expectoration of thick, yellow mucus. Pulsatilla has a similar hoarseness, but the expectoration consists of yellow, thick, or greenish mucus, and the expectoration is only present during the morning or during the day. Another great difference exists between Kali bichr. and Pulsat., not only in modifying their curative power in this disease, but in general, for Kali bichr. has an aggravation of almost all its symptoms from cold—open cold air—while Pulsatilla has an amelioration from the same causes.

During the prevalence of epidemic measles I have seen frequent occurrence of cases that, as soon as the eruption developed itself, a hoarse, croupy cough set in, which much distressed the patient, the nose became sore, small ulcers formed in the nostrils, and the discharge became at once tough, thick, viscid, stiff. The expectoration soon became stringy and tough, and detached with great difficulty; the patient was then generally much worse than in the ordinary measles. Aconite, which corresponds with the slight, intolerable, hacking cough, preventing sleep and causing great restlessness, gave no relief, nor did Bry. or Phosph. control this cough, but Kali bichr., which I always give in the two hundredth or a higher potency, promptly cured these cases, and the patients generally recovered fully without any other medicine. Kali bichr. is not unfrequently the true homœopathic remedy in croup—when the expectoration is tough, stringy, and ropy.

Whenever this ropy, stringy condition of the mucus prevails, Kali bichr. will be often the only curative remedy.* While the presence of these symptoms may often lead us to give Kali bichr. in croup, as well as in diphtheria, or in bronchitis, there are other symptoms often present which will call our attention to this medicine. In February last a very violent case of diphtheria, made worse by a relapse from overheating and then running out in the cold air, in a child five years old, which had been convalescent under the action of Lachesis, was finally cured by Kali bichr. The symptoms indicating it were "violent stitches in the left ear, extending into the roof of the mouth, into the corresponding side of the head, and into the same side of the neck, which was painful to the touch, and the glands swollen." This symptom prevailed, *and had appeared last;* the swelling on the neck was larger than a goose-egg; it finally suppurated and discharged under the influence of Kali bichr., while all diphtheritic symptoms gradually improved, and the child fully recovered without any further medication.

In ozæna, we find Kali bichr. at times the curative remedy. We find the following symptoms: Nose full of thick mucus; profuse discharge of thick, clear mucus; if that ceases, he has pain going from the occiput to the forehead; considerable discharge of mucus without having coryza; *discharge from the nose of hard plugs, called by the workmen clinkers, elastic, like India-rubber;* the pain in the nose at the junction of the cartilage; discharge from the nose of hard, greenish-colored masses, some-

---

* In hooping-cough, when the mucus is abundant, threatening suffocation, white and stringy, Coccus cacti is indicated.

times of a disagreeable smell. All of these symptoms are often present in ozæna; the cessation of the discharge, especially when it is sudden, often causes violent headache, which has often been relieved at once by one dose of Kali bichr.$^{200}$, and the habitual discharge restored. A case of chronic ozæna was much improved, where the following symptom indicated Kali bichr.; this symptom promptly disappeared, and the chronic disease was also much lessened. "Violent shooting pains from the root of the nose along the left orbital arch to the external angle of the eye exactly, with dimness of sight, like a scale before the eye; *beginning in the morning, it increases till noon, and gives way toward the evening.*"

In this case I had my attention called to Kali bichr. first by the periodicity of the daily recurring paroxysms of pain always increasing from morning till twelve M. All the other symptoms also corresponding with Kali bichr. The patient, a lady of forty-six years of age, received one dose of Kali bichr.$^{200}$, and was soon relieved of the paroxysms; the discharge from the nose was also much diminished.

In the so-called dyspepsia, we find Kali bichr. often to be the proper medicine. After a meal, which had been enjoyed, a sensation as if digestion was impeded, and the food rested on the stomach like a heavy weight. Kali bichr. will relieve this symptom often. Nux vom. has something very similar; but the difference between the two remedies is, that the heavy weight and pressure on the stomach is felt under Kali bichr. at once after a meal, while under Nux vom. sometimes one to three hours elapse before it is felt.

I was first induced to give Kali bichr. for the bad effects of over-indulgence in beer and other malt liquors, as well for the acute results as also for the chronic ailments of the habitual beer-drinkers, by the following symptoms. In the morning nausea and sensation of heaviness in the head and eyes. Nausea when walking about. Nausea and *vomiting of mucus.* Occasional attacks of indigestion; loss of appetite, the food presses like a heavy weight; bad humor; suffering much from flatulency; *in the morning he feels confused, has nausea,* and sometimes vomits a clear fluid. A very frequent complaint of those who indulge habitually and freely in malt liquors, is a great weight in the pit of the stomach; flatulency, loss of appetite; and when they eat, the food oppresses them at once; nausea; confused feeling, especially in the morning, *and vomiting of mucus.* When these symptoms presented themselves, Kali bichr.$^{200}$ has always cured promptly. Other results of the overindulgence in malt liquors, especially ale, are diseases of the liver; they also often find their remedy in Kali bichr.

In many cases where the round ulcer of the stomach could readily be diagnosticated, Kali bichr. was an important remedy, provided its symptoms otherwise corresponded with those of the patient.

Kali bichr. is applicable in secondary syphilis. Long-continued erythematous blush of the fauces and soft palate, varying in hue from a dark to a bright red, occasionally of a copper color. On the right side of the root of the uvula, an excavated sore, half the size of a split pea, with a reddish areola, and containing a yellow, tenacious matter; fauces and palate presenting an erythematous blush. Uvula and tonsils become red, swollen, and painful, and finally ulcerate; this caused a surgeon to believe it to be syphilitic. Guided by these symptoms, I have administered Kali bichr. in the not unfrequent syphilitic ulceration of the throat, and also for that less frequent, but very dangerous ulcer which appears on the root of the uvula, and which sometimes destroys the uvula in less than three days, finally extending to the soft palate, where the destruction is rapid. It has also cured syphilitic ulcers of the tongue; the indication for its use has been painful ulcer on the tongue, which lasted for weeks.

Clinical observations have shown that the stringy, tough condition of the mucus is not confined to the secretions and discharges from the respiratory organs; but by analogy it has been administered in and permanently cured fluor albus when the discharge was stringy and tough.

Kali bichr. is also beneficial in some forms of dysentery. Dysenteric attacks, with pains about the navel and bloody evacuations. Shortly after dinner, sudden nausea, sensation of pressure in the region of the stomach; pricking, pinching pain about the liver; urging to vomit; rumbling in the lower abdomen; discharge of very offensive flatulence; violent pinching in the whole abdomen, cutting as if the abdomen was lacerated with knives in all directions; after a discharge of fæces of the usual consistency, seven to eight dysenteric discharges of brown, frothy water, with voilent, painful pressing, urging, and tenesmus in the anus; nausea; desire to vomit and pain in the abdomen. For many years, toward the beginning of summer, was subject to an attack of dysentery, lasting three weeks. Frequent bloody alvine evacuations with gnawing pain in the naval region, followed by unsuccessful urgings; tongue smooth, red, becomes cracked; urging to stool; collection of water in the mouth and nausea; burning pain in the anus and erections continuing over half an hour. Pressing in the anus, and tenesmus in the sphincter ani.

Clinical experiences have verified these symptoms; some permanent cures have been made in cases of dysentery returning periodically every year in the early part of the summer, which attacks not only yielded at once to Kali bichr., but did not return the following years. The red, smooth, and cracked tongue in dysentery is characteristic of Kali bichr.

The ulcers for which Kali bichr. is most curative are large, with a dark centre and overhanging edges.

